# Retraction: Eco-friendly soil amendments improve growth, antioxidant activities, and root colonization in lingrain (*Linum Usitatissimum* L.) under drought conditions

**DOI:** 10.1371/journal.pone.0272534

**Published:** 2022-08-17

**Authors:** The PLOS ONE Editors

The *PLOS ONE* Editors retract this article [1] because it was identified as one of a series of submissions for which we have concerns about authorship, competing interests, and peer review. We regret that the issues were not addressed prior to the article’s publication.

HH, RA, ATKZ, and RZS did not agree with the retraction. MF and AHG either did not respond directly or could not be reached.
